# Cellular and extracellular miRNAs are blood‐compartment‐specific diagnostic targets in sepsis

**DOI:** 10.1111/jcmm.13162

**Published:** 2017-04-06

**Authors:** Marlene Reithmair, Dominik Buschmann, Melanie Märte, Benedikt Kirchner, Daniel Hagl, Ines Kaufmann, Martina Pfob, Alexander Chouker, Ortrud K. Steinlein, Michael W. Pfaffl, Gustav Schelling

**Affiliations:** ^1^ Institute of Human Genetics University Hospital, Ludwig‐Maximilians‐University Munich Germany; ^2^ Division of Animal Physiology and Immunology TUM School of Life Sciences Weihenstephan Technical University Munich Munich Germany; ^3^ Department of Anesthesiology University Hospital, Ludwig‐Maximilians‐University Munich Germany; ^4^ Department of Anaesthesiology Neuperlach Hospital City Hospitals of Munich Munich Germany

**Keywords:** sepsis, exosome, blood compartment, liquid biopsy, miRNA, biomarker

## Abstract

Septic shock is a common medical condition with a mortality approaching 50% where early diagnosis and treatment are of particular importance for patient survival. Novel biomarkers that serve as prompt indicators of sepsis are urgently needed. High‐throughput technologies assessing circulating microRNAs represent an important tool for biomarker identification, but the blood‐compartment specificity of these miRNAs has not yet been investigated. We characterized miRNA profiles from serum exosomes, total serum and blood cells (leukocytes, erythrocytes, platelets) of sepsis patients by next‐generation sequencing and RT‐qPCR (*n* = 3 × 22) and established differences in miRNA expression between blood compartments. *In silico* analysis was used to identify compartment‐specific signalling functions of differentially regulated miRNAs in sepsis‐relevant pathways. In septic shock, a total of 77 and 103 miRNAs were down‐ and up‐regulated, respectively. A majority of these regulated miRNAs (14 in serum, 32 in exosomes and 73 in blood cells) had not been previously associated with sepsis. We found a distinctly compartment‐specific regulation of miRNAs between sepsis patients and healthy volunteers. Blood cellular miR‐199b‐5p was identified as a potential early indicator for sepsis and septic shock. miR‐125b‐5p and miR‐26b‐5p were uniquely regulated in exosomes and serum, respectively, while one miRNA (miR‐27b‐3p) was present in all three compartments. The expression of sepsis‐associated miRNAs is compartment‐specific. Exosome‐derived miRNAs contribute significant information regarding sepsis diagnosis and survival prediction and could serve as newly identified targets for the development of novel sepsis biomarkers.

## Introduction

Sepsis has been described as one of the oldest and most pressing problems in medicine 1. Even with treatment in modern intensive care units (ICUs), mortality rates of patients with septic shock are in the range between 30% and 44.3% 2 with an even higher in‐hospital mortality of up to 50% 3.

Pathobiological models of sepsis emphasize the important role of host immunity in disease development. Current theories postulate that organ injury and death from sepsis are the result of an unabated activation of innate‐immunity‐driven inflammation accompanied by a down‐regulation of genes that modulate the adaptive immune response. On the other hand, there is clear evidence for a prolonged phase of immunosuppression.

Despite these controversies, there is a consensus that rapid cell‐to‐cell communication between the immune system and afflicted organs plays an important role during the cascade of events leading from initial contact with an invasive pathogen to the fulminant clinical syndrome of septic shock.

A growing body of literature suggests that nano‐sized extracellular vesicles, including exosomes, play a critical role in cell‐to‐cell communication, especially in inflammatory processes and malignancy 4. Interestingly, small non‐coding microRNAs (miRNAs) are highly enriched in exosomes and certain miRNAs are over‐represented compared to their fraction in the donor cell 5, 6.

These miRNAs play a crucial role in exosome‐mediated phenotypic modulation of recipient cells. It is well known that miRNAs are capable of influencing gene expression by specifically binding to the 3′‐untranslated region (UTR) of complementary mRNA sequences and thus repressing their translation. Previous publications were able to demonstrate that exosomes from platelets under septic insult induce myocardial dysfunction and apoptosis in endothelial cells, and may thus contribute to the vascular abnormalities commonly observed in patients with sepsis 7, 8. Consequently, cell‐to‐cell delivery of miRNAs through circulating exosomes could represent an important mechanism of long‐range signalling during sepsis and offers promising opportunities for assessing biomarkers in minimally invasive liquid biopsies.

In this study, we examined serum‐derived exosomes from patients with septic shock and compared their miRNA expression levels to those from total serum and blood cells (leukocytes, erythrocytes, platelets) using comprehensive high‐throughput next‐generation sequencing (NGS).

## Materials and methods

### Patient recruitment and sample collection

In total, 22 critically ill patients with sepsis were included in the study and matched to 23 healthy volunteers (Table S1). Patients were recruited from two anaesthesiological ICUs caring for a mixed medical/surgical patient population of an academic medical centre of the University of Munich and a city hospital of Munich.

Healthy volunteers were recruited from hospital personnel and by advertisement.

Seven patients with septic shock were initially selected for NGS and matched to seven age and gender comparable healthy volunteers. RT‐qPCR confirmation of expression levels of selected miRNAs was performed in another 15 patients with varying disease severity and in a matched control group of equal size.

Blood samples were drawn from healthy subjects *via* venipuncture and from patients through intravascular catheters on day 0 and day 4 (if available). Samples to obtain serum were collected into 9 ml serum tubes (S‐Monovette; Sarstedt AG&Co, Nümbrecht, Germany), immediately centrifuged at 3400 × *g* for 10 min. and frozen. Whole blood samples designated for extraction of cellular miRNAs were collected in RNA tubes (PAXgene; Qiagen, Hilden, Germany) according to the manufacturer's protocol. Serum aliquots and RNA tubes were stored at −80°C.

### Exosome isolation and quality control

**Figure 1:**
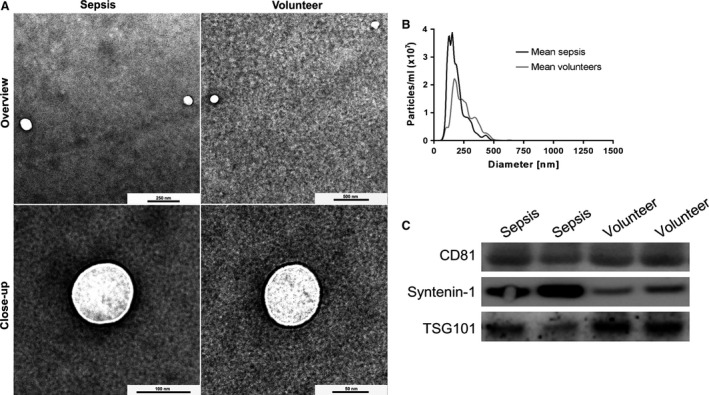
Exosome characterization by transmission electron microscopy (TEM), nanoparticle tracking analysis (NTA) and WB. TEM revealed vesicles with an average size range of about 100–150 nm (**A**). NTA showed a sharp size distribution with a mean particle diameter of 154.4 ± 40.2 nm for sepsis patients (black) and 225.2 ± 24.3 nm for volunteers (grey) (**B**). Exosomes were detected by WB using the exosomal markers CD81, TSG101 and syntenin‐1 (**C**).

3 ml serum was digested with 34 μl thrombin for 5 min. After centrifugation (10,000 × *g*, 5 min.), exosomes were isolated from the supernatant using miRCURY™ Exosome Isolation Kit according to the manufacturer's protocol (Exiqon A/S). The presence and purity of exosomes were confirmed by transmission electron microscopy (TEM) and nanoparticle tracking analysis (NTA). Exosomal marker proteins (CD81, TSG101, syntenin‐1) were substantiated by immunoblotting (Fig. 1).

For TEM, exosomes were diluted in PBS and deposited on formvar‐coated copper grids (Plano GmbH, Wetzlar, Germany) for 60 sec. Grids were negative stained in 2% uranyl acetate (Sigma Aldrich Co., Taufkirchen, Germany) before imaging on a Zeiss EM900 (Carl Zeiss Microscopy GmbH, Munich, Germany) with a wide‐angle dual‐speed 2K‐CCD camera at 80 kV.

For NTA, exosomes were analysed using a NanoSight LM10 instrument (Malvern Instruments GmbH, Malvern, UK) equipped with a monochromatic 405 nm laser and a high‐sensitivity sCMOS camera. Exosome preparations were diluted in PBS and recorded by the NTA software (Malvern Instruments GmbH, Malvern, UK). Five videos of 30 sec. with a frame rate of 25 frames/sec were recorded. Each particle was subsequently tracked on a frame‐by‐frame basis by the Finite Track Length Adjustment algorithm, and the recorded Brownian motion was used to infer particle sizes by applying the Stokes–Einstein equation.

For Western blot analysis (WB), miRCURY™ precipitates were washed in PBS and pelleted by ultracentrifugation at 100,000 × *g* for 2 hrs (Beckman Coulter Optima LE‐80K using a SW60 Ti rotor, k‐factor: 167.9, 4°C). The pellets were lysed in ice‐cold RIPA buffer with a protease inhibitor cocktail (Roche Deutschland Holding GmbH, Grenzach‐Wyhlen, Germany). Lysates were sonicated, and then centrifuged at 13,000 × *g* for 5 min., and protein concentration in the supernatant was measured using a BCA assay (Sigma Aldrich) before separation by SDS‐PAGE. Lysates were boiled in reducing sample buffer for 10 min., and fractionated using NuPAGE 4–12% Bis‐Tris Gels (Invitrogen, Carlsbad, California, USA). Proteins were transferred to a nitrocellulose membrane (GE Healthcare Life Sciences, Freiburg, Germany). Primary antibodies were from antibodies‐online.com (rabbit anti‐syntenin‐1, ABIN1881779, 1:1000) or OriGene Technologies, Inc., Rockville, Maryland, USA (rabbit anti‐CD81, TA343281, 1:1000 and rabbit anti‐TSG101, TA343598, 1:500). Secondary antibodies were from Santa Cruz Biotechnology, Dallas, Texas, USA (goat anti‐rabbit IgG‐HRP, sc‐2030, 1:5000).

### Extraction of extracellular and cellular RNA

After exosome isolation from 3 ml serum, exosomal RNA was extracted with the miRCURY™ RNA Isolation Kit—Biofluids (Exiqon, Vedbaek, Denmark) and eluted in 30 μl nuclease‐free water.

Serum of 600 μl were extracted with the miRCURY™ RNA Isolation Kit—Biofluids (Exiqon) according to the manufacturer's protocol. RNA was eluted in 30 μl nuclease‐free water.

For extraction of blood cell RNA, PAXgene blood tubes were processed with the PAXgene blood miRNA kit (Qiagen) according to the manufacturer's protocol. Integrity of total blood cell‐derived RNA was assessed with the RNA 6000 Nano assay on the Bioanalyzer 2100 (Agilent Technologies, Inc., Santa Clara, California, USA). Small RNA fractions in cellular and extracellular samples were analysed using the Small RNA assay and RNA yield for cell‐derived RNA was quantified on the Qubit 2.0 Fluorometer with the RNA HS Assay Kit (Life Technologies, Carlsbad, California, USA) according to the manufacturer's protocol.

### Calculation of haemolysis scores

Blondal *et al*. 9 found that in RT‐qPCR a delta Cq of miR‐23a–miR‐451a >7–8 indicates a high risk for haemolysis bias in the samples. Haemolysis was assessed for each patient sample by calculating the Cq ratio of these miRNAs accordingly. All samples included in the study showed a delta Cq < 7 and thus a low risk for haemolysis.

### Next‐generation sequencing

Blood cell, exosomal and serum RNA from seven patients diagnosed with septic shock as well as seven healthy volunteers were sequenced for small RNA.

Blood cell‐derived RNA of 100 ng was used as starting material, while the entire RNA yields from 3 ml, or 600 μl serum were used for exosomal RNA and serum RNA, respectively. Library preparation was performed as described in Spornraft *et al*. 10. Libraries were subsequently subjected to Illumina single‐end sequencing by synthesis, using 50 cycles on the HiSeq 2500 (Illumina Inc., San Diego, California, USA).

Sequencing data were processed as described previously 10. A log_2_ fold change ≥|1| and an adjusted p value (Padj) of ≤0.05 were set as thresholds to identify significantly regulated miRNAs. Only transcripts with a baseMean ≥50 were included in the analysis. For technical NGS data, see Figure S1.

### Bioinformatic analysis

Ingenuity Pathway Analysis (IPA^®^) (Qiagen) was used for the identification of causal networks in our high‐throughput miRNA expression data. In our analyses, we only considered pathways with a negative log *P* value >20. Only miRNAs meeting the pre‐defined cut‐off values mentioned above were entered into IPA^®^, and only experimentally confirmed or highly predicted relationships were considered for the analysis of miRNA effects.

Significantly regulated miRNAs exclusively present in one of the compartments (exosomes, serum and blood cells) but not in any of the others were entered into *IPA* ^*®*^ *microRNA Target Filter* to identify target genes of relevance to sepsis. For this purpose, disease filtering was set to *Antimicrobial Response*,* Inflammatory Response* and *Infectious Disease*. miRNAs from all three compartments were then included in a *Core Analyses* step, which identified pathways and causal networks regulated within each compartment. In a final step, *IPA* ^*®*^ *Comparison Analysis* was used to generate heat maps to visualize and compare the canonical pathways and disease states of relevance to miRNAs regulated within the three compartments.

### RT‐qPCR validation

In all, 20 miRNAs covering baseMeans from 69 to 279,000 were investigated by RT‐qPCR in a new cohort of 15 septic patients on day 0 (= day of hospitalization) and day 4, as well as 16 healthy volunteers. geNorm 11 and NormFinder 12 were used to predict the most stable miRNAs for each of the three compartments based on the NGS data set, and six potential reference miRNA candidates were selected. For RT‐qPCR, 10 ng of blood cell‐derived RNA was reverse transcribed with the universal cDNA synthesis kit II according to the manufacturer's protocol (Exiqon). Exosomal and serum RNA were normalized to the volume of starting material (3 ml serum and 600 μl serum, respectively), and 2 μl of a 30 μl eluate were subjected to reverse transcription. RT‐qPCR was performed in a 10 μl total reaction volume of ExiLENT SYBR^®^ Green master mix and 1 μl of each miRNA LNA™ PCR primer (Exiqon). PCR reactions were performed on a Rotor‐Gene Q (Qiagen) or CFX384 (Bio‐Rad) real‐time cycler. Relative quantification was carried out using the ΔΔCq method 13.

### Statistical analysis

Demographic characteristics between volunteers and sepsis patients were compared using analysis of variance (anova) with *post‐hoc* testing. For comparison of demographic and clinical parameters between the NGS group and the RT‐qPCR confirmation sample, Student′s *t*‐test was applied in case of normally distributed data and the Wilcoxon rank‐sum test for non‐parametric continuous variables. The Chi‐square or Fisher′s exact test was used for comparison of categorical variables. Data in the text and in tables are given as mean ± SD. To increase clarity in some of the figures, NGS and RT‐qPCR data are expressed as mean ± SEM. Statistical calculations were performed using SPSS (version 24.0, IBM GmbH, Ehningen, Germany).

### Ethics approval and consent to participate

The study was approved by the Ethics Committee of the Medical Faculty of the University of Munich (protocol #551‐14). The study was carried out according to the World Medical Association Declaration of Helsinki and all study samples were anonymized during analysis.

Written informed consent for publication of blinded individual personal data was obtained from each participant or the patient's legal representative.

## Results

### Exosome isolation and characterization

WB analysis revealed the presence of exosome markers CD81, TSG101 and syntenin‐1. Single particle analysis by NTA revealed a narrow size distribution with mean particle diameters of 154.4 ± 40.2 nm (mode: 110.7 ± 23.5 nm) and 225.2 ± 24.3 nm (mode: 169.7 ± 21.8 nm) for septic patients and healthy volunteers, respectively. No significant differences in particle number were observed between the two groups. TEM revealed a homogeneous population, no morphological differences were observed in vesicles from healthy volunteers and sepsis patients (Fig. 1).

### Differential gene expression analysis

For each of the three analysed compartments, miRNA reads were compared between septic shock patients and healthy volunteers. Compared to healthy volunteers, 77 distinct miRNAs were down‐regulated, whereas 103 distinct miRNAs were up‐regulated in septic shock patients. The majority of regulated miRNAs was found exclusively in blood cell‐derived samples and amounted to 62.14% for up‐regulated and 64.94% for down‐regulated miRNAs, respectively. The extracellular compartment accounted for 23.38% of all down‐regulated (exosome: 14.29%; serum: 3.90%; both: 5.19 %) and 28.16% of all up‐regulated (exosome: 15.53%; serum: 3.88%; both: 8.74%) miRNAs. A small fraction of miRNAs was found to be simultaneously up‐ or down‐regulated in all three compartments (2.91% and 3.90%, respectively) (Table S2).

### Newly identified sepsis‐associated miRNAs

The literature was searched for previously sepsis‐associated miRNAs and compared to significantly regulated miRNAs from this study. For this purpose, each miRNA was searched for in PubMed in combination with the terms ‘sepsis’; ‘lipopolysaccharide’ and ‘lps’. miRNAs with ambiguous literature results were considered as PubMed hits and thus categorized as previously sepsis‐associated. Applying the mentioned criteria, our NGS dataset provided 32 newly identified sepsis‐associated miRNAs in exosomes, 14 in serum and 73 in blood cells (Table S2). Seventeen of these miRNAs were found exclusively in exosomes, respectively, four and 67 miRNAs in serum or blood cells. One miRNA, miR‐423‐5p, was present in all three compartments, showing down‐regulation in the sepsis shock cohort.

### RT‐qPCR validation

**Figure 2:**
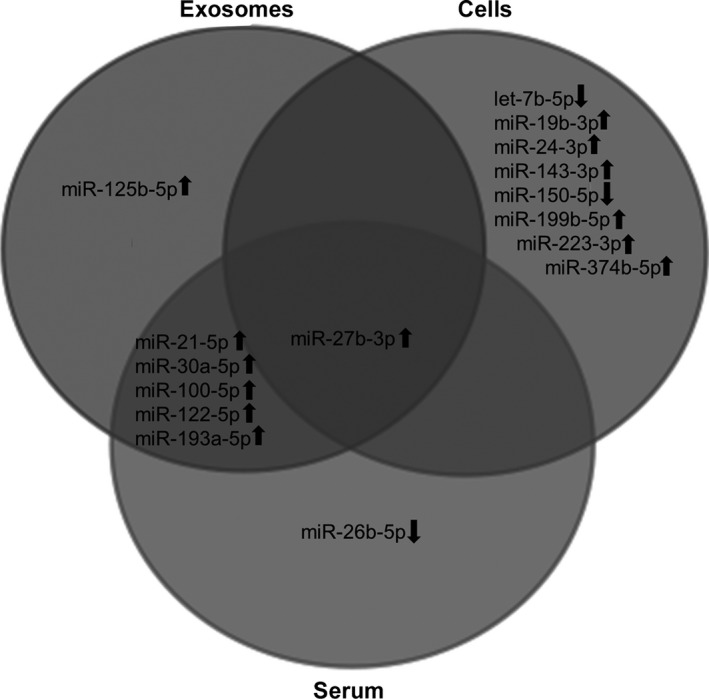
Venn diagram of differential miRNA expression profiles from septic shock patients validated by RT‐qPCR.

Subsequent to normalization with the geometric mean of the reference miRNAs miR‐185‐5p and miR‐25‐3p, differential miRNA expression between healthy volunteers and patients with sepsis or septic shock were assessed. For exosome and serum samples, seven out of 12 miRNAs (58.3%) selected from NGS results could be validated in RT‐qPCR, while nine out of 14 miRNAs (64.3%) were validated for cellular samples. Log_2_ fold changes of validated miRNAs for septic shock patients in NGS (*n* = 7) and RT‐qPCR (*n* = 6) were correlated for each compartment (Fig. S2). One of the validated miRNAs was present in all compartments while one, one and eight were unique to exosomes, serum and blood cells, respectively. Five miRNAs were present in both extracellular compartments, but not in blood cells (Fig. 2).

### Disease detection by miRNAs

**Figure 3:**
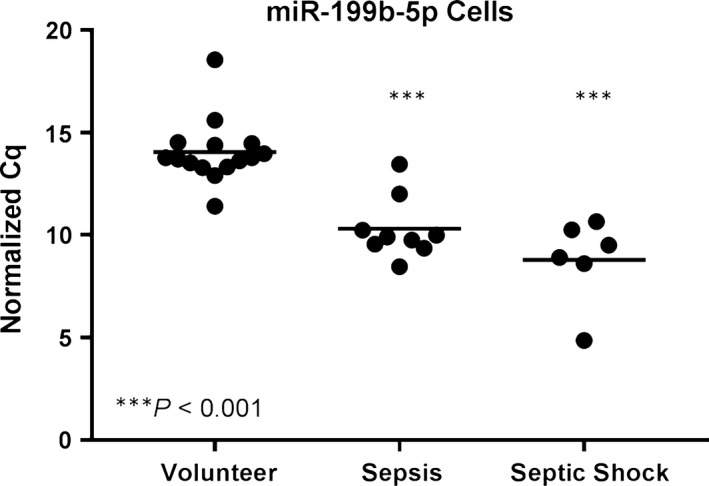
Sepsis detection by miRNAs: Only cellular miR‐199b‐5p detected both sepsis and septic shock with high confidence (*n* = 6).

RT‐qPCR data were screened for miRNAs distinguishing patients with both sepsis and septic shock from healthy volunteers on the day of admission to the ICU. One miRNA, cellular miR‐199b‐5p, also indicated the less severe stage with high confidence (*P* < 0.001) (Fig. 3).

### miRNA expression levels across sepsis stages

**Figure 4:**
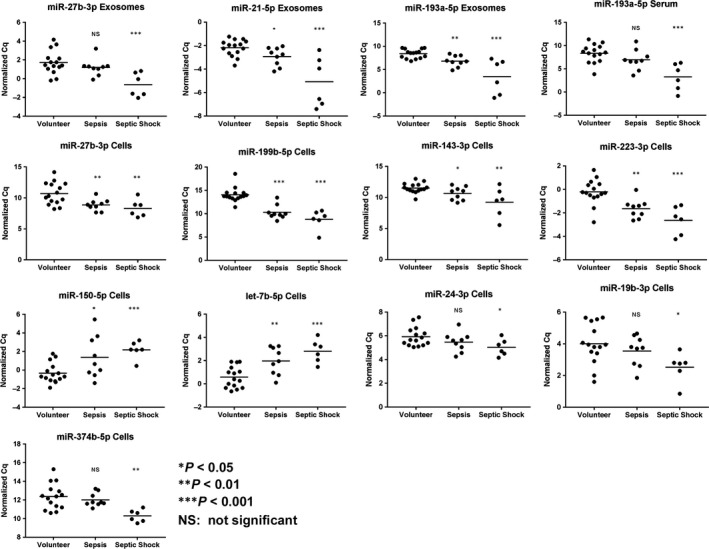
Relationship between miRNA expression level and disease severity. Eleven miRNAs in at least one compartment display stringent correlation of normalized Cq values and disease severity in septic patients upon admission to the ICU.

In order to assess potential regulations of miRNAs across disease stages, expression levels from healthy volunteers (*n* = 16) and patients at study inclusion (day 0) suffering from sepsis (*n* = 9) and septic shock (*n* = 6) were compared. Almost linear correlations of expression level and disease severity were found for 11 different miRNAs in at least one compartment (three exosomal, one serum, nine cellular) (Fig. 4). With the exception of cellular miR‐150‐5p and let‐7b‐5p, all miRNAs correlating to disease severity were up‐regulated. Two miRNAs showed correlations in two compartments: miR‐27b‐3p was simultaneously up‐regulated in exosomes and blood cells from septic patients, while miR‐193a‐5p was exclusively up‐regulated in extracellular samples. All 11 miRNAs showed significant differences (*P* < 0.05) in expression levels between the healthy state and septic shock. Additionally, multiple miRNAs in exosomes (miR‐21‐5p, miR‐193a‐5p), serum (miR‐26b‐5p) and cells (let‐7b‐5p, miR‐27b‐3p, miR‐143‐3p, miR‐150‐5p, miR‐199b‐5p, miR‐223‐3p) were also able to differentiate (*P* < 0.05) between healthy volunteers and septic patients (Fig. S3).

### Survival prediction by extracellular miRNAs

**Figure 5:**
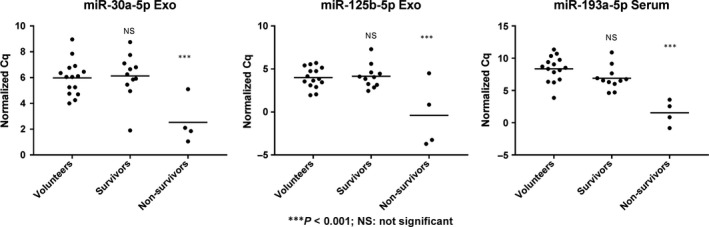
Survival prediction by miRNAs. Exosomal miR‐30a‐5p and miR‐125b‐5p and serum miR‐193a‐5p predict survival of septic patients with high confidence.

RT‐qPCR patients were grouped according to outcome (death, *n* = 4; survival, *n* = 11) irrespective of sepsis stage. On the day of admission to the ICU, three extracellular miRNAs (exosomal miR‐30a‐5p and miR‐125b‐5p, miR‐193a‐5p in serum) were able to predict survival with high confidence (*P* < 0.001) (Fig. 5). Septic shock was markedly associated with death (75.0% of non‐survivors and 27.3% of survivors were characterized to be in septic shock) with significantly different expression levels between disease stages for miR‐193a‐5p and miR‐125b‐5p.

### Longitudinal analysis of miRNAs correlating with disease stage

**Figure 6:**
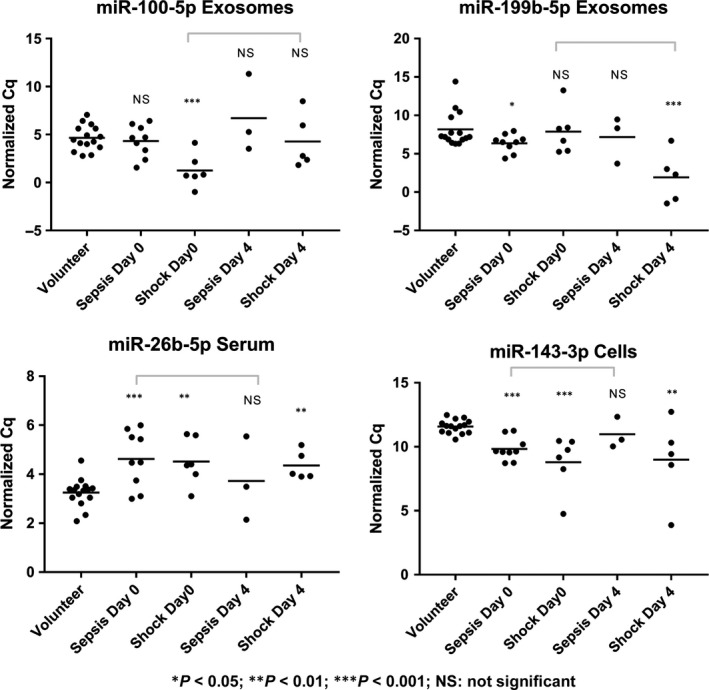
Significantly different miRNA expression levels between days 0 and 4. Samples on day 4 were available from eight of the patients with sepsis or septic shock previously used in RT‐qPCR expression analysis at day 0. Grey bars indicate notable changes in expression within a group of patients. Tested miRNAs were exosomal miR21‐5p, miR27b‐3p, miR100‐5p, miR193a‐5p, miR199b‐5p, serum miR26b‐5p and miR193a‐5p, as well as blood cellular miR27b‐3p, miR143‐3p, miR150‐5p, miR199b‐5p, miR223‐3p and let‐7b‐5p.

Expression levels of selected miRNAs on day 0 and day 4 were assessed by RT‐qPCR in a separate experiment on patients with sepsis (*n* = 3) and septic shock (*n* = 5). Some miRNAs showed a normalization of expression levels on day 4 (miR‐100‐5p exosomes, miR‐26b‐5p serum, miR‐143‐3p cells), while exosomal miR‐199b‐5p was found to become more up‐regulated compared to healthy volunteers (*n* = 16) (Fig. 6).

### *In silico* analyses of differentially expressed miRNAs

Top canonical pathways identified by the target disease‐driven approach mentioned above were *Pathogen Influenced Signalling, Cellular and Humeral Immune Response* including *Cytokine Signalling, Cellular Stress Response and Injury* and *Cardiovascular Signalling* (Fig. S4).

## Discussion

There are a number of earlier studies that have investigated miRNAs in the context of sepsis. However, only two studies took a genome‐wide approach for assessing the complete miRNA expression profile in human patients with sepsis in comparison with non‐septic controls by means of NGS 14, 15. Another study by Vasilescu *et al*. 16 profiled miRNAs derived from peripheral blood leukocytes by microarray analysis and validated the most dysregulated miRNA in their sepsis cohort, miR‐150, in plasma samples from septic patients. Although their clinical study used a less sensitive approach (microarray instead of NGS), it is the only one investigating at least one miRNA in both cellular and extracellular blood compartments in human sepsis patients. In light of the scarcity of miRNA profiling studies in patients with this disorder, and taking into consideration the limited possibility of inter‐study comparison due to different compartments, it is essential to comprehensively profile the blood miRNA signature of critically ill patients based on all compartments.

Comprehensive small RNA‐Seq of blood compartments in septic individuals, performed in this study, revealed compartment‐specific differences in miRNA profiles. While exosomes and serum were found to share a significant number of miRNAs, there was very limited overlap in the miRNA profiles of cellular and extracellular samples. Individual sampling compartments in blood thus differ not only in RNA quantity, but also display qualitatively distinct profiles of bioactive nucleic acids. Particularly, exosomes, released by various cell populations in the body and thus present in the extracellular blood fraction, play a major role as miRNA carriers and mediators of intercellular communication 17, 18, 19. Considered an enriched sampling fraction, the exosomal miRNA profile may serve as an excellent source of diagnostic biomarkers for identifying sepsis patients early on, discriminating different sepsis stages or even predicting the course of the disease. In this context, it is worth mentioning that, to our knowledge, no clinical study characterizing miRNAs derived from exosomes as a miRNA‐relevant compartment in blood has been carried out in sepsis patients before.

When profiling miRNAs differentially regulated in sepsis patients on the day of admission to the ICU by RT‐qPCR, only one blood cell‐derived miRNA (miR‐199b‐5p) distinguished both sepsis and septic shock from healthy volunteers. Conversely, the miRNAs significantly associated with survival (miR‐30a‐5p, miR‐125b‐5p, miR‐193a‐5p) were found to be extracellular. Due to this non‐uniform distribution of miRNA information across sample types, it seems prudent to choose the appropriate blood compartment for the respective question at hand.

In our data, tracking miRNAs in patients upon admission to the ICU and 96 hrs afterwards revealed varying courses of regulation which were, again, compartment specific. While expression of some up‐/down‐regulated miRNAs normalized over time, others were found to deviate even further from healthy volunteers. This might be due to heterogeneous patient responses to clinical care, differences in type of treatment and medication received or a combination thereof. Serial measurements of compartment‐specific miRNAs might therefore also hold value for sepsis management.

As a conclusion, this study presents the first comprehensive profiling of cellular and extracellular miRNAs in sepsis patients. We found a distinctly compartment‐specific regulation of miRNAs between sepsis patients and healthy volunteers. NGS‐based profiling allowed the identification of sepsis patients as opposed to healthy volunteers, prognosis of patient survival and elucidation of miRNA disease functions. Additionally, we detected multiple compartment‐specific, highly regulated miRNAs that had not previously been associated with sepsis. Most importantly, our data demonstrate the benefit of including exosomal miRNAs in disease assessment protocols. The practicality and applicability of sampling miRNAs in liquid biopsies will enhance biomarker research and eventually the clinical management of sepsis.

## Conflict of interest

The authors confirm that there are no conflicts of interest.

## Supporting information


**Figure S1** Technical NGS quality and quantity data for each matrix.

**Figure S2** Correlation of NGS and RT‐qPCR log2 fold changes for validated miRNAs.

**Figure S3** miRNAs differentiating between healthy volunteers and sepsis patients.

**Figure S4** Heat maps for the 5 Top Canonical Pathway networks.

**Table S1** Summary of demographic and clinical characteristics of study groups.

**Table S2** Significantly regulated miRNAs in septic shock patients detected by NGS.
