# Supplementary material for: Cellular and extracellular miRNAs are blood‐compartment‐specific diagnostic targets in sepsis
Source: J Cell Mol Med. 2017 Apr 6;21(10):2403–11. doi: 10.1111/jcmm.13162 (PMC5618677; doi:10.1111/jcmm.13162)
Supplement: Supplementary file 1 — Figure S1 Technical NGS quality and quantity data for each matrix. [file JCMM-21-2403-s001.pdf]

Supplemental Figure 1. Technical NGS quality and quantity data for each matrix.

Fractions of small RNA from exosomes, serum and blood cells were analyzed by small RNA-Seq using Illumina sequencing by synthesis chemistry. Sequencing septic shock patients resulted in mean library sizes of $3.94 \times 10^6 \pm 4.22 \times 10^6$ reads for exosomal, $5.01 \times 10^6 \pm 4.62 \times 10^6$ reads for serum and $2.67 \times 10^7 \pm 1.37 \times 10^7$ reads for cellular samples. Library sizes for healthy volunteers were $7.65 \times 10^6 \pm 6.32 \times 10^6$ from exosomes, $5.48 \times 10^6 \pm 4.46 \times 10^6$ from serum and $2.16 \times 10^7 \pm 9.78 \times 10^6$ from cells. Overall sequencing quality was high, with a mean per base Phred score of 36.33 ± 0.26 (SEM) and a mean per sequence Phred score of 35.94 ± 0.09 (SEM).. Trimmed reads were subsequently filtered by depleting sequences pertaining to rRNA, tRNA, snRNA and snoRNA, and matched to known miRNAs by alignment to miRBase. Mean reads mapping to human miRNAs for septic shock patients were: $2.99 \times 10^6 \pm 2.67 \times 10^6$ (exosomes), $9.99 \times 10^5 \pm 1.85 \times 10^6$ (serum), $2.50 \times 10^6 \pm 1.29 \times 10^6$ (blood cells); for volunteers: $1.66 \times 10^6 \pm 1.20 \times 10^6$ (exosomes), $3.16 \times 10^5 \pm 1.95 \times 10^5$ (serum) and $2.54 \times 10^6 \pm 1.13 \times 10^6$ (blood cells).

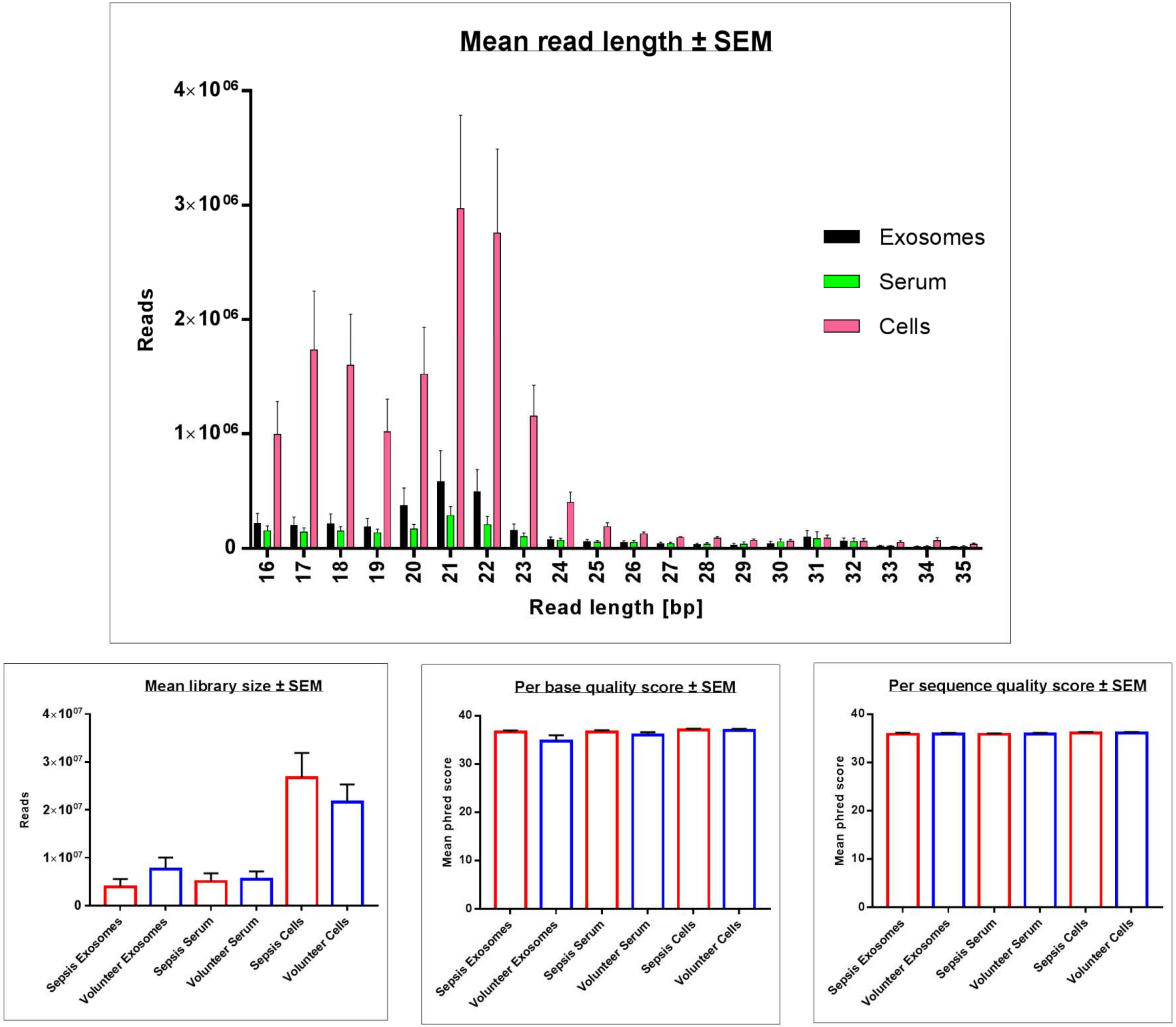

Mean read length ± SEM
Reads
4×10^06
3×10^06
2×10^06
1×10^06
0
16 17 18 19 20 21 22 23 24 25 26 27 28 29 30 31 32 33 34 35
Read length [bp]
Exosomes
Serum
Cells
Mean library size ± SEM
Reads
4×10^07
3×10^07
2×10^07
1×10^07
0
Sepsis Exosomes
Volunteer Exosomes
Sepsis Serum
Volunteer Serum
Sepsis Cells
Volunteer Cells
Per base quality score ± SEM
Mean phred score
40
30
20
10
0
Sepsis Exosomes
Volunteer Exosomes
Sepsis Serum
Volunteer Serum
Sepsis Cells
Volunteer Cells
Per sequence quality score ± SEM
Mean phred score
40
30
20
10
0
Sepsis Exosomes
Volunteer Exosomes
Sepsis Serum
Volunteer Serum
Sepsis Cells
Volunteer Cells
