# Supplementary material for: Cellular and extracellular miRNAs are blood‐compartment‐specific diagnostic targets in sepsis
Source: J Cell Mol Med. 2017 Apr 6;21(10):2403–11. doi: 10.1111/jcmm.13162 (PMC5618677; doi:10.1111/jcmm.13162)

Supplemental Figure 2. Correlation of NGS and RT-qPCR log_2 fold changes for validated miRNAs. miRNAs for RT-qPCR were chosen from NGS results based on the following criteria: same direction of regulation in all three compartments; only significantly regulated extracellularly (exosomes and serum); only significantly regulated cellularly counter-regulated in extracellular versus cellular compartments.

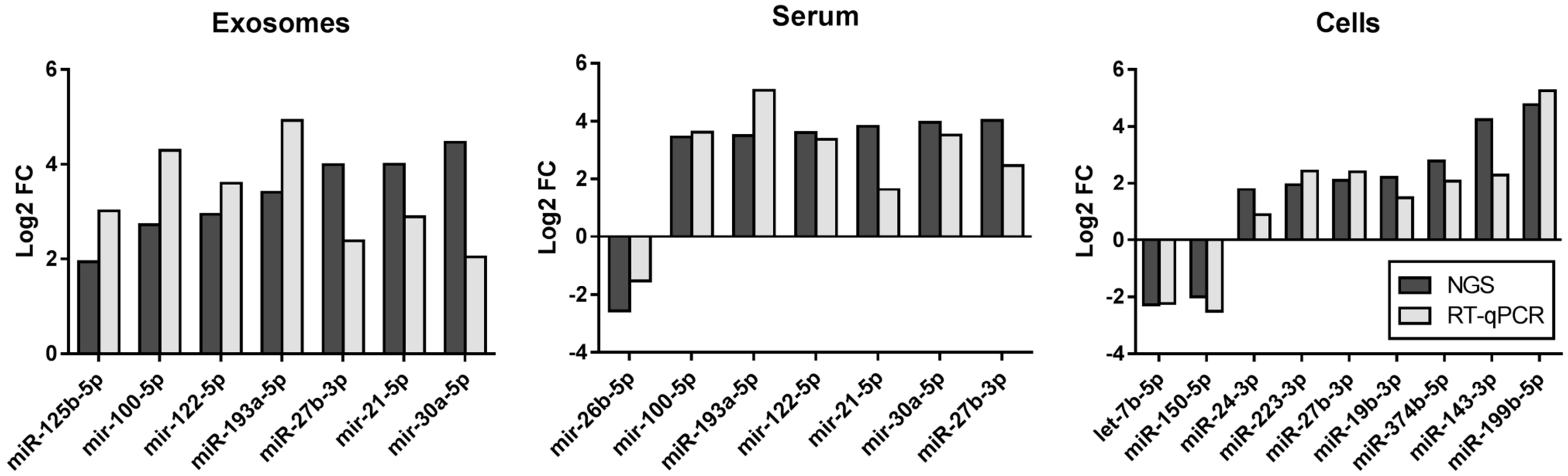

Supplement: Supplementary file 2 — Figure S2 Correlation of NGS and RT‐qPCR log2 fold changes for validated miRNAs. [file JCMM-21-2403-s002.pdf]
