# Supplementary material for: Cellular and extracellular miRNAs are blood‐compartment‐specific diagnostic targets in sepsis
Source: J Cell Mol Med. 2017 Apr 6;21(10):2403–11. doi: 10.1111/jcmm.13162 (PMC5618677; doi:10.1111/jcmm.13162)

Supplemental Figure 3. miRNAs differentiating between healthy volunteers and sepsis patients. Multiple miRNAs in exosomes, serum and cells were also able to differentiate ($p<0.05$) between healthy volunteers and sepsis patients.

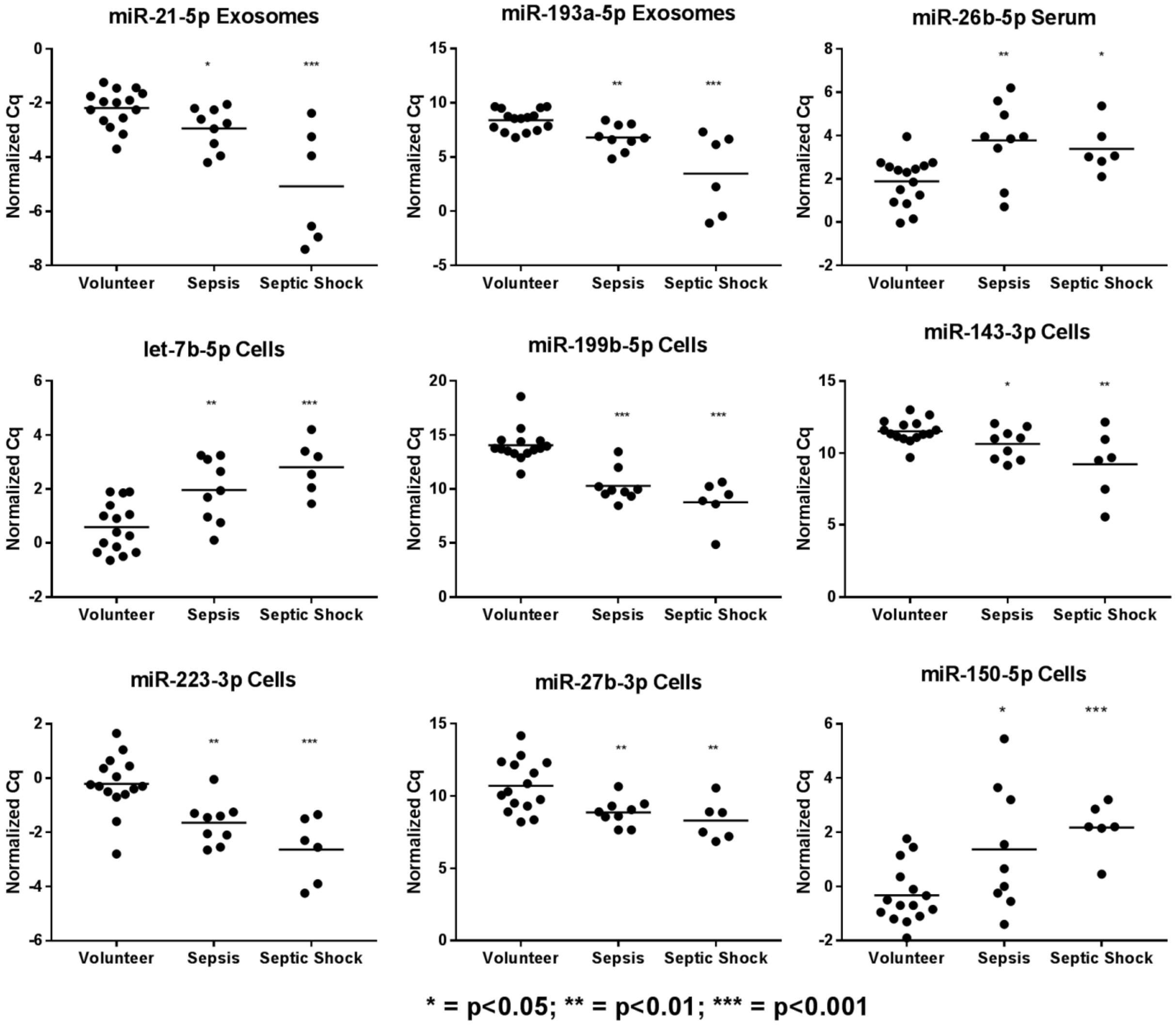

Supplement: Supplementary file 3 — Figure S3 miRNAs differentiating between healthy volunteers and sepsis patients. [file JCMM-21-2403-s003.pdf]
