# Supplementary material for: Cellular and extracellular miRNAs are blood‐compartment‐specific diagnostic targets in sepsis
Source: J Cell Mol Med. 2017 Apr 6;21(10):2403–11. doi: 10.1111/jcmm.13162 (PMC5618677; doi:10.1111/jcmm.13162)

Supplemental Figure 4. Heat maps for the 5 Top Canonical Pathway networks. Increasing IPA® pathway scores, which represent the negative log p-value, are depicted by increased shadowing of the rectangles of the heat map.

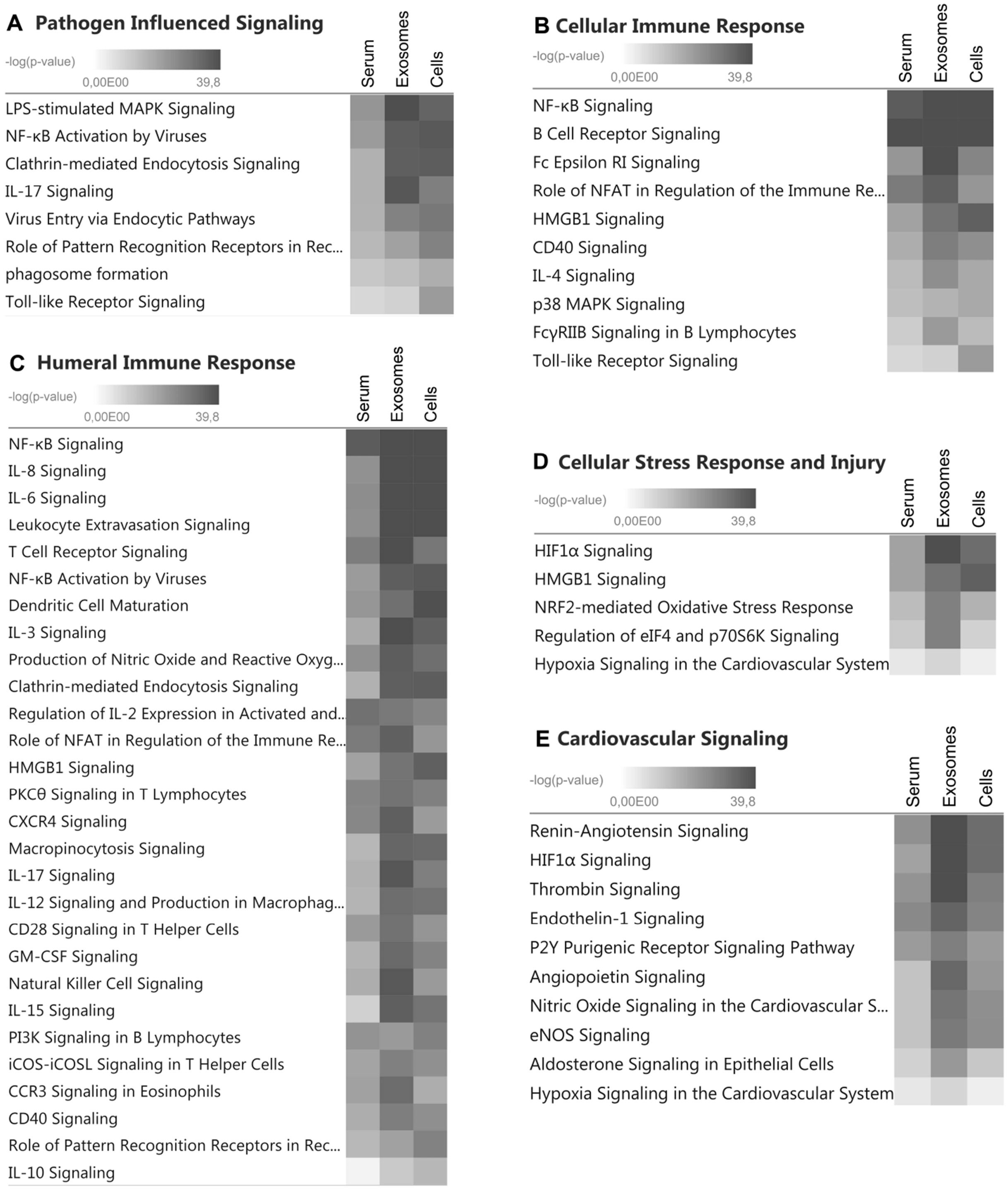

Supplement: Supplementary file 4 — Figure S4 Heat maps for the 5 Top Canonical Pathway networks. [file JCMM-21-2403-s004.pdf]
