# Supplementary material for: Cellular and extracellular miRNAs are blood‐compartment‐specific diagnostic targets in sepsis
Source: J Cell Mol Med. 2017 Apr 6;21(10):2403–11. doi: 10.1111/jcmm.13162 (PMC5618677; doi:10.1111/jcmm.13162)
Supplement: Supplementary file 5 — Table S1 Summary of demographic and clinical characteristics of study groups. [file JCMM-21-2403-s005.pdf]

Supplemental Table 1. Summary of demographic and clinical characteristics of study groups. Patients were included in the study if they were > 18 years of age and were within 24 h after admission to the ICU. Exclusion criteria were pregnancy, immunosuppression, leukopenia, hematological malignancies, or the initiation of palliative care. Only volunteers with a Charlson Comorbidity Index of ≤ 1 [1] were included as controls.

**Variable**	**Sepsis - NGS[a] (n=7)**	**Sepsis – RT-qPCR[b] confirmation (n=15)**	**Healthy volunteers NGS (n=7)**	**Healthy volunteers RT-qPCR[b] (n=16)**
Male/female gender (n)	6/1	9/6	4/3	6/10
Age (years)	54.6 ± 6.9	69.6 ± 19.1*	51.0 ± 7.90	53.9 ± 11.1
Weight (kg)	85.7 ± 21.0	77.8 ± 14.4	72.9 ± 13.0	73.2 ± 13.0
Height (cm)	177.0 ± 8.1	171.3 ± 7.0	172.3 ± 9.5	171.0 ± 8.9
Body mass index (BMI)	27.3 ± 4.9	27.0 ± 5.0	24.3 ± 2.6	25.0 ± 2.9
Septic Shock (n)	7[§]	6		
Source of infection (n)			n/a	n/a
Pneumonia	3	1		
Urinary tract		1		
Other/unknown	4	13		
Organism (n)			n/a	n/a
Gram-positive bacteria	4	7		
Mixed		6		
Gram-negative bacteria		1		
Unknown	2	1		
Other	1			
Mechanical ventilation (n)	7[+]	6	n/a	n/a
Acute pulmonary injury (n) (ARDS[c])	5	4	n/a	n/a
Acute kidney injury[d] (n)	5	9	n/a	n/a
AKIN score[e]	1.3±1.3	1.3±1.3	n/a	n/a
Apache II[f] score	25.6±4.3[++]	13.5±9.3	n/a	n/a
SOFA[g] score	14.1±3.0[++]	5.8±4.1	n/a	n/a
Serum lactate levels (mmol/l)	8.7±8.7	4.9±7.9[h]	n/a	n/a

ICU length-of-stay (days)	34.0±25.8[#]	10.3±12.8	n/a	n/a
Hospital length-of-stay (days)	42.4±31.5	25.3±13.6	n/a	n/a
Death (n)	3	4	n/a	n/a

[a]original sample; [b]confirmatory samples; [c]Acute Respiratory Distress Syndrome (defined according to Berlin criteria [2]); [d]Defined according to Acute Kidney Injury Network (AKIN) criteria [3]; [e]Acute Kidney Injury Network Staging System for Acute Kidney Injury classification system with a range of 1 to 3 with higher scores indicating more severe injury [3]; [f]Acute Physiology and Chronic Health Evaluation II is a severity-of-disease classification system for critically ill patients. Scores range from 0 to 71, higher scores correspond to more severe disease and a higher risk of death [4]; [g]Sequential Organ Failure Assessment (SOFA) score is a scoring system to determine the extent of a person's organ function or rate of failure. Scores less than 9 indicate predictive mortality at 33% while above 11 can be close to or above 95% [5]; [h]Initial lactate measurements at admission to the ICU were missing in 3 patients from the RT-qPCR group; *indicates significantly higher patient age in the sepsis RT-qPCR group as compared to healthy volunteers ($p \leq 0.033$); [+]significantly more patients from the NGS – group required mechanical ventilation during ICU therapy compared to the RT-qPCR group ($p = 0.017$); [§]all patients from the NGS group had septic shock whereas 6/15 patients from the RT-qPCR group had the condition ($p = 0.010$); [++]significantly higher Apache II and SOFA scores in patients from the NGS group ($p = 0.004$ and $p < 0.001$, respectively); [#]significantly longer duration of ICU-therapy in patients from the NGS group ($p = 0.008$). n, number; n/a, not applicable. Data are mean ± SD.

1. **Charlson ME, Pompei P, Ales KL, MacKenzie CR.** A new method of classifying prognostic comorbidity in longitudinal studies: development and validation. *J Chronic Dis*. 1987; 40: 373-83.

2. **Ferguson ND, Fan E, Camporota L, Antonelli M, Anzueto A, Beale R, Brochard L, Brower R, Esteban A, Gattinoni L, Rhodes A, Slutsky AS, Vincent JL, Rubenfeld GD, Thompson BT, Ranieri VM.** The Berlin definition of ARDS: an expanded rationale, justification, and supplementary material. *Intensive care medicine*. 2012; 38: 1573-82.

3. **Lopes JA, Fernandes P, Jorge S, Goncalves S, Alvarez A, Costa e Silva Z, Franca C, Prata MM.** Acute kidney injury in intensive care unit patients: a comparison between the RIFLE and the Acute Kidney Injury Network classifications. *Critical care (London, England)*. 2008; 12: R110.

4. **Knaus WA, Draper EA, Wagner DP, Zimmerman JE.** APACHE II: a severity of disease classification system. *Crit Care Med*. 1985; 13: 818-29.

5. **Vincent JL, Moreno R, Takala J, Willatts S, De Mendonca A, Bruining H, Reinhart CK, Suter PM, Thijs LG.** The SOFA (Sepsis-related Organ Failure Assessment) score to describe organ dysfunction/failure. On behalf of the Working Group on Sepsis-Related Problems of the European Society of Intensive Care Medicine. *Intensive care medicine*. 1996; 22: 707-10.
