# Supplementary material for: Cellular and extracellular miRNAs are blood‐compartment‐specific diagnostic targets in sepsis
Source: J Cell Mol Med. 2017 Apr 6;21(10):2403–11. doi: 10.1111/jcmm.13162 (PMC5618677; doi:10.1111/jcmm.13162)
Supplement: Supplementary file 6 — Table S2 Significantly regulated miRNAs in septic shock patients detected by NGS. [file JCMM-21-2403-s006.pdf]

Supplemental Table 2. Significantly regulated miRNAs in septic shock patients detected by NGS.

	miRNA	Log_2FC[a]	Padj[b]
**Upregulated in exosomes**			
	[d]hsa-miR-21-3p	1.60	0.0314
	hsa-miR-499a-5p	1.93	0.0152
	[d]hsa-miR-125b-5p	1.94	0.0425
	hsa-miR-424-5p	2.02	0.0092
	hsa-miR-542-3p	***2.19***	***0.0146***
	hsa-miR-378a-5p	2.35	0.0321
	[d]hsa-miR-30e-5p	***2.48***	***0.0343***
	hsa-miR-497-5p	***2.49***	***0.0039***
	[d]hsa-miR-148a-5p	***2.58***	***0.0057***
	[d]hsa-miR-30a-3p	2.59	0.0068
	[c]hsa-miR-100-5p	***2.72***	***0.0156***
	hsa-miR-30d-3p	***2.75***	***0.0111***
	hsa-miR-335-5p	***2.76***	***0.0029***
	[c]hsa-miR-3591-3p	***2.78***	***0.0257***
	hsa-miR-375	***2.83***	***0.0251***
	hsa-miR-34a-5p	2.88	0.0216
	[f]hsa-miR-122-5p	2.94	0.0184
	hsa-miR-125b-2-3p	***3.02***	***0.0146***
	hsa-miR-95-3p	***3.06***	***5.25E-03***
	hsa-miR-885-5p	***3.29***	***9.28E-03***

	[c]hsa-miR-193a-5p	***3.40***	***3.74E-04***
	[c]hsa-miR-99a-5p	3.46	4.49E-03
	hsa-miR-146b-3p	3.54	9.62E-03
	hsa-miR-193b-5p	3.62	4.48E-03
	[c]hsa-miR-210-3p	3.62	2.07E-03
	[c]hsa-miR-378d	***3.70***	***2.88E-03***
	[d]hsa-miR-148a-3p	***3.84***	***2.07E-03***
	hsa-miR-548ah-3p	***3.84***	***4.49E-03***
	[c]hsa-miR-378c	***3.94***	***1.68E-03***
	[d]hsa-miR-194-5p	3.99	3.26E-03
	[f]hsa-miR-27b-3p	3.99	2.07E-03
	[f]hsa-miR-21-5p	4.00	1.67E-03
	[c]hsa-miR-30a-5p	4.46	6.10E-04
	[c]hsa-miR-452-5p	***5.63***	***7.45E-06***
**Upregulated in serum**			
	[e]hsa-miR-192-5p	3.21	0.0472
	hsa-miR-22-3p	***3.34***	***0.0464***
	[c]hsa-miR-100-5p	***3.46***	***0.0343***
	[c]hsa-miR-3591-3p	***3.60***	***0.0464***
	[f]hsa-miR-122-5p	3.61	0.0472
	[c]hsa-miR-193a-5p	***3.63***	***0.0003***
	hsa-miR-619-5p	***3.64***	***0.0245***
	[c]hsa-miR-99a-5p	3.65	0.0245
	[e]hsa-miR-21-5p	3.83	0.0312

	[c]hsa-miR-210-3p	3.88	0.0024
	[c]hsa-miR-30a-5p	3.96	0.0120
	[f]hsa-miR-27b-3p	4.03	0.0207
	hsa-miR-378a-3p	4.07	0.0028
	[c]hsa-miR-378d	***4.23***	***0.0080***
	hsa-miR-4488	***4.31***	***0.0017***
	[c]hsa-miR-378c	***4.45***	***0.0050***
	[c]hsa-miR-452-5p	***6.06***	***4.09E-04***
**Upregulated in blood cells**			
	hsa-miR-330-3p	1.01	0.0325
	hsa-miR-23a-3p	1.14	0.0205
	hsa-miR-106b-3p	1.17	0.0264
	hsa-miR-421	1.20	0.0256
	hsa-miR-532-5p	***1.22***	***0.0084***
	hsa-miR-7-5p	***1.23***	***0.0186***
	hsa-miR-103a-3p	1.23	0.0353
	hsa-miR-339-3p	***1.27***	***0.0350***
	hsa-miR-4677-3p	***1.27***	***0.0184***
	hsa-miR-4690-3p	***1.28***	***0.0410***
	hsa-miR-106b-5p	1.29	0.0141
	hsa-miR-3688-3p	***1.30***	***0.0258***
	hsa-miR-3909	***1.32***	***0.0045***
	hsa-miR-183-5p	***1.38***	***0.0296***
	hsa-miR-581	***1.43***	***0.0220***

	hsa-miR-181a-3p	1.43	0.0052
	hsa-miR-340-3p	***1.44***	***0.0144***
	hsa-miR-16-5p	1.49	0.0095
	hsa-miR-503-5p	1.53	0.0100
	hsa-let-7g-5p	1.55	0.0233
	[d]hsa-miR-21-3p	1.56	0.0307
	hsa-let-7f-5p	1.57	0.0073
	hsa-miR-98-5p	1.59	0.0182
	hsa-miR-15b-3p	1.61	0.0003
	hsa-miR-30e-3p	1.61	0.0009
	hsa-miR-10b-5p	***1.63***	***0.0002***
	hsa-miR-142-5p	***1.65***	***0.0042***
	hsa-miR-182-5p	1.66	0.0011
	[f]hsa-miR-21-5p	1.71	0.0054
	hsa-miR-362-5p	***1.74***	***0.0070***
	hsa-miR-146b-5p	1.78	0.0014
	hsa-miR-3074-5p	***1.79***	***0.0070***
	hsa-miR-144-5p	***1.81***	***0.0353***
	hsa-miR-152-3p	***1.85***	***0.0016***
	[d]hsa-miR-30a-3p	1.87	1.01E-04
	[d]hsa-miR-30e-5p	***1.88***	***3.15E-04***
	hsa-miR-99b-5p	***1.95***	***0.0212***
	hsa-miR-1248	***1.96***	***0.0049***
	[e]hsa-miR-192-5p	1.99	3.69E-04
	[e]hsa-miR-223-3p	1.99	0.0025

	hsa-miR-24-3p	2.04	0.0014
	hsa-miR-148b-3p	2.06	6.13E-05
	hsa-miR-15b-5p	2.07	0.0020
	hsa-miR-660-5p	***2.09***	***4.03E-05***
	[d]hsa-miR-194-5p	2.09	1.57E-05
	hsa-miR-96-5p	2.10	3.33E-03
	hsa-miR-618	***2.12***	***3.04E-03***
	hsa-miR-1290	2.16	2.79E-03
	hsa-miR-769-5p	***2.16***	***2.50E-03***
	hsa-miR-363-3p	***2.19***	***3.74E-04***
	hsa-miR-451a	2.22	7.08E-07
	[f]hsa-miR-27b-3p	2.22	0.0230
	[e]hsa-miR-26a-5p	2.27	2.75E-05
	hsa-miR-450a-5p	***2.27***	***1.49E-04***
	hsa-miR-145-5p	2.29	0.0027
	hsa-miR-19b-3p	2.29	3.89E-04
	hsa-miR-548o-3p	***2.31***	***4.81E-05***
	hsa-miR-29b-3p	2.33	1.49E-04
	hsa-miR-1246	***2.34***	***4.71E-04***
	[d]hsa-miR-148a-5p	***2.36***	***0.0014***
	hsa-miR-29a-3p	2.43	1.23E-06
	hsa-miR-142-3p	2.47	0.0025
	hsa-miR-215-5p	2.54	6.59E-06
	[d]hsa-miR-374b-5p	2.78	2.71E-06
	hsa-miR-19a-3p	3.11	1.64E-05

	hsa-let-7a-3p	3.19	2.71E-06
	hsa-let-7f-2-3p	3.28	2.75E-05
	[d]***hsa-miR-148a-3p***	***3.41***	***2.39E-09***
	hsa-miR-548e-3p	***3.47***	***2.31E-07***
	[f]hsa-miR-122-5p	3.59	2.15E-05
	hsa-miR-340-5p	***3.89***	***2.24E-11***
	hsa-miR-143-3p	4.45	1.76E-14
	hsa-miR-199b-5p	***5.12***	***1.01E-14***
	hsa-miR-582-3p	***6.25***	***4.67E-15***
**Downregulated in exosomes**			
	[c]***hsa-miR-4448***	***-4.36***	***2.38E-04***
	[f]hsa-let-7b-5p	-3.52	7.45E-06
	hsa-miR-323b-3p	***-3.35***	***0.0065***
	[f]hsa-let-7d-5p	-3.35	0.0049
	hsa-miR-4497	***-3.20***	***0.0065***
	hsa-miR-654-3p	***-3.15***	***0.0185***
	hsa-miR-543	***-2.83***	***0.0314***
	[d]***hsa-miR-370-3p***	***-2.81***	***0.0407***
	hsa-miR-409-3p	***-2.41***	***0.0040***
	[f]***hsa-miR-423-5p***	***-2.38***	***9.48E-04***
	[c]***hsa-miR-3184-3p***	***-2.35***	***0.0021***
	hsa-miR-432-5p	***-2.27***	***0.0274***
	hsa-miR-4792	***-2.27***	***0.0188***
	hsa-miR-493-5p	***-2.25***	***0.0201***

	[d]hsa-miR-342-5p	-2.21	0.0093
	[d]hsa-let-7c-5p	-2.04	0.0041
	[c]hsa-miR-744-5p	***-1.94***	***0.0057***
	[d]hsa-let-7a-5p	-1.93	0.0333
	hsa-let-7e-5p	-1.91	0.0403
	[d]hsa-miR-342-3p	-1.86	0.0400
	[d]hsa-miR-374b-5p	-1.82	0.0045
	hsa-miR-18a-5p	-1.79	0.0212
	[c]hsa-miR-26b-5p	-1.49	0.0120
	[d]hsa-miR-320a	***-1.44***	***0.0138***
**Downregulated in serum**			
	[f]hsa-miR-423-5p	***-3.78***	***3.44E-06***
	[f]hsa-let-7d-5p	-3.45	0.0058
	[e]hsa-miR-223-3p	-3.23	0.0024
	[c]hsa-miR-4448	***-3.17***	***0.0472***
	hsa-miR-199a-5p	***-3.04***	***0.0274***
	[f]hsa-let-7b-5p	-3.04	2.98E-04
	[e]hsa-miR-26a-5p	-2.71	0.0233
	[c]hsa-miR-26b-5p	-2.56	0.0336
	[c]hsa-miR-744-5p	***-2.45***	***0.0245***
	[c]hsa-miR-3184-3p	***-1.85***	***0.0419***
**Downregulated in blood cells**			
	hsa-miR-485-3p	***-2.95***	***0.0025***

	hsa-miR-16-2-3p	***-2.82***	***2.97E-11***
	[f]hsa-let-7b-5p	-2.74	1.58E-04
	hsa-miR-150-5p	-2.33	2.71E-06
	hsa-miR-4732-3p	***-2.25***	***4.68E-09***
	hsa-let-7i-5p	-2.22	4.00E-03
	hsa-miR-3605-3p	***-2.19***	***2.31E-07***
	hsa-miR-3615	***-2.19***	***4.63E-06***
	hsa-miR-6833-3p	***-2.18***	***1.32E-04***
	hsa-miR-574-5p	-2.13	2.15E-05
	hsa-miR-4685-3p	***-2.12***	***1.00E-03***
	hsa-miR-1275	***-2.10***	***6.42E-04***
	hsa-miR-6803-3p	***-2.06***	***3.01E-04***
	hsa-miR-1306-5p	***-2.04***	***0.0101***
	hsa-miR-6511a-3p	***-2.00***	***7.15E-05***
	hsa-miR-3940-3p	***-1.98***	***0.0116***
	hsa-miR-92b-3p	-1.96	2.50E-03
	hsa-miR-6857-3p	***-1.94***	***0.0016***
	hsa-miR-326	***-1.93***	***4.35E-05***
	hsa-miR-320d	***-1.92***	***4.36E-05***
	hsa-miR-6747-3p	***-1.92***	***1.70E-05***
	hsa-miR-4301	***-1.92***	***1.95E-03***
	[d]hsa-miR-320a	***-1.90***	***3.29E-06***
	hsa-miR-328-3p	***-1.90***	***8.02E-06***
	hsa-miR-6511b-3p	***-1.89***	***1.33E-04***
	hsa-miR-3173-5p	-1.87	4.52E-07

	hsa-miR-320b	***-1.85***	***6.59E-06***
	hsa-miR-210-5p	-1.84	2.23E-03
	hsa-miR-6786-3p	***-1.80***	***2.39E-04***
	hsa-miR-92a-3p	-1.75	2.53E-03
	hsa-miR-1976	***-1.75***	***1.51E-04***
	hsa-miR-486-5p	-1.73	4.03E-05
	hsa-miR-636	***-1.71***	***6.45E-05***
	hsa-miR-6741-3p	***-1.71***	***9.48E-04***
	hsa-miR-766-3p	***-1.68***	***0.0107***
	[d]hsa-let-7a-5p	-1.64	0.0217
	hsa-miR-320c	***-1.64***	***7.19E-05***
	hsa-miR-4732-5p	***-1.63***	***0.0230***
	hsa-miR-6131	***-1.62***	***0.0126***
	hsa-miR-942-5p	***-1.61***	***0.0011***
	[d]hsa-miR-342-3p	-1.56	0.0207
	[d]hsa-miR-342-5p	-1.53	0.0263
	[f]hsa-let-7d-5p	-1.48	0.0215
	hsa-miR-126-3p	-1.48	0.0346
	[d]hsa-let-7c-5p	-1.46	0.0480
	hsa-miR-574-3p	***-1.44***	***0.0042***
	hsa-miR-5010-3p	***-1.42***	***0.0095***
	[d]hsa-miR-125b-5p	-1.41	0.0182
	hsa-miR-1249-3p	***-1.38***	***0.0019***
	hsa-miR-6783-3p	***-1.30***	***0.0058***
	hsa-miR-937-3p	***-1.29***	***0.0081***

	hsa-miR-93-3p	-1.29	0.0052
	[d]***hsa-miR-370-3p***	***-1.20***	***0.0295***
	hsa-miR-18a-3p	-1.17	7.74E-04
	hsa-miR-2110	***-1.13***	***0.0122***
	hsa-miR-532-3p	***-1.12***	***0.0045***
	hsa-miR-6842-3p	***-1.08***	***0.0180***
	[f]***hsa-miR-423-5p***	***-1.06***	***0.0106***
	hsa-miR-296-5p	***-1.02***	***0.0084***

[a]Log_2 fold change (Log_2FC) of $\geq |1|$; [b]adjusted p-value (Padj) of ≤ 0.05; [c]miRNA only present extracellularly in exosome and serum; [d]miRNA present in exosome and blood cells; [e]miRNA present in serum and blood cells; [f]miRNA present in all three matrices; bold italic: newly identified sepsis-associated miRNA candidates
